# Embodied inference and spatial cognition

**DOI:** 10.1007/s10339-012-0519-z

**Published:** 2012-07-29

**Authors:** Karl Friston

**Affiliations:** The Wellcome Trust Centre for Neuroimaging, Institute of Neurology, University College London, Queen Square, London, WC1N 3BG UK

**Keywords:** Free energy, Active inference, Visual search, Surprise, Salience, Exploration, Embodied cognition, Perception

## Abstract

How much about our interactions with—and experience of—our world can be deduced from basic principles? This paper reviews recent attempts to understand the self-organised behaviour of embodied agents, like ourselves, as satisfying basic imperatives for sustained exchanges with the environment. In brief, one simple driving force appears to explain many aspects of perception, action and the perception of action. This driving force is the minimisation of surprise or prediction error, which—in the context of perception—corresponds to Bayes-optimal predictive coding (that suppresses exteroceptive prediction errors) and—in the context of action—reduces to classical motor reflexes (that suppress proprioceptive prediction errors). In what follows, we look at some of the phenomena that emerge from this single principle, such as the perceptual encoding of spatial trajectories that can both generate movement (of self) and recognise the movements (of others). These emergent behaviours rest upon prior beliefs about itinerant (wandering) states of the world—but where do these beliefs come from? In this paper, we focus on the nature of prior beliefs and how they underwrite the active sampling of a spatially extended sensorium. Put simply, to avoid surprising states of the world, it is necessary to minimise uncertainty about those states. When this minimisation is implemented via prior beliefs—about how we sample the world—the resulting behaviour is remarkably reminiscent of searches seen in foraging or visual searches with saccadic eye movements.

## Introduction

If perception corresponds to hypothesis testing (Gregory 1980), then visual searches could correspond to experiments that generate sensory data. In this paper, we explore the idea that saccadic eye movements are optimal experiments, in which data are gathered to test hypotheses or beliefs about how those data are caused. This provides a plausible model of visual search that can be motivated from the basic principles of self-organised behaviour—namely the imperative to minimise the entropy of hidden states of the world and their sensory consequences. This imperative is met if agents sample hidden states of the world efficiently. This efficient sampling of salient information can be derived in a fairly straightforward way, using information theory and approximate Bayesian inference. Simulations of the resulting active inference scheme reproduce sequential eye movements that are reminiscent of empirically observed saccades and provide some counterintuitive insights into the way that sensory evidence is accumulated or assimilated into beliefs about the world.

## Active inference and the free energy principle

We start with the assumption that biological systems minimise the dispersion or entropy of states in their external milieu—to ensure a sustainable and allostatic exchange with their environment (Ashby 1947). Clearly, these states are hidden and cannot be measured or changed directly. However, if agents know how their action changes sensations—for example, if they know contracting certain muscles will necessarily excite primary sensory afferents from stretch receptors—then they can minimise the dispersion—or entropy—of their sensory states by countering surprising deviations from expected values. If the uncertainty about hidden states, given sensory states, is small, then this minimisation of sensory entropy through action will be sufficient to minimise the entropy of hidden states. In this setting, entropy corresponds to average surprise or uncertainty. However, minimising surprise through action is not as straightforward as it might seem, because measuring surprise is almost impossible. This is where *variational free energy* comes in—to provide an upper bound on surprise that enables agents to minimise free energy instead of surprise. However, in using an upper bound on surprise, the agent now has to minimise the difference between surprise and the free energy by changing its internal states. This corresponds to Bayes-optimal perception (Yuille and Kersten 2006) and associates internal brain states with conditional or posterior representations of hidden states in the world (Helmholtz 1866/1962; Gregory 1980; Ballard et al. 1983; Dayan et al. 1995; Friston 2005).

## Predictive coding and action

**Fig. 1:**
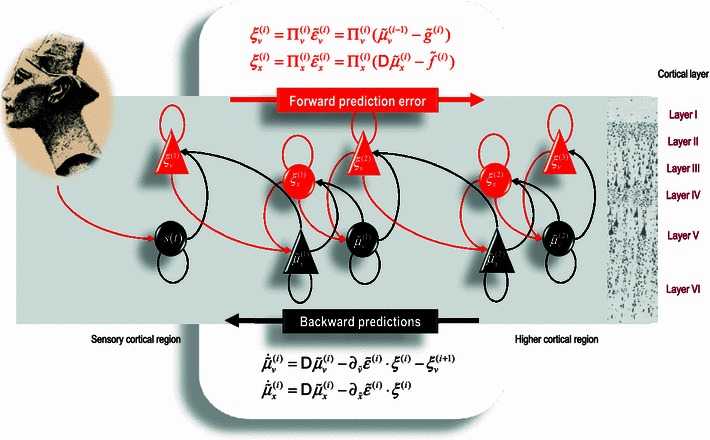
Schematic detailing the neuronal architectures that might encode posterior expectations about the states of a hierarchical generative model. This figure shows the speculative cells of origin of forward driving connections that convey prediction error from a lower area to a higher area and the backward connections that construct predictions (Mumford 1992). These predictions try to explain away prediction error in lower levels. In this scheme, the sources of forward and backward connections are superficial and deep pyramidal cells, respectively. The equations represent a generalised descent on free energy under the hierarchical models described in Friston (2008). State units are in *black* and error units in *red*. Here, neuronal populations are deployed hierarchically within three cortical areas (or macrocolumns). Within each area, the cells are shown in relation to cortical layers: supragranular (I–III) granular (IV) and infragranular (V–VI) layers

Neurobiological implementations of free energy minimisation are known as predictive coding and have become a popular framework for understanding message passing in the brain—see Fig. 1. In the present context, one can regard free energy as the amplitude of prediction errors, so that minimising free energy means optimising predictions—encoded by internal brain states—to suppress prediction errors. Clearly, to make predictions, the brain has to have a model, or hypothesis, and explaining how sensory input was generated: this is known as a *generative model*.

**Fig. 2:**
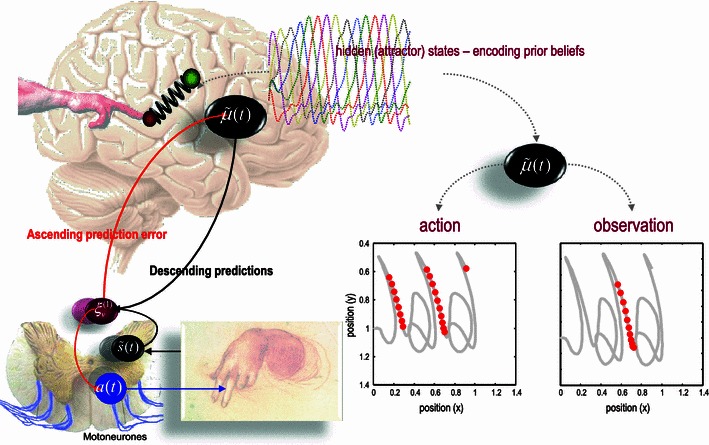
This schematic summarizes the results of the simulations of action observation reported in Friston et al. (2011). The *left panel* pictures the brain as a forward or generative model of itinerant movement trajectories (based on a Lotka-Volterra attractor, whose states are shown as a function of time in *coloured lines*). This model furnishes predictions about visual and proprioceptive inputs, which prescribe movement through reflex arcs at the level of the spinal cord (insert on the *lower left*). The variables have the same meaning as in the previous figure. The mapping between attractor dynamics and proprioceptive consequences is modelled with Newtonian mechanics on a two jointed arm, whose extremity (*red ball*) is drawn to a target location (*green ball*) by an imaginary spring. The location of the target is prescribed (in an extrinsic frame of reference) by the currently active state in the attractor. These attractor dynamics and the mapping to an extrinsic (movement) frame of reference constitute the agent’s prior beliefs. The ensuing posterior beliefs are entrained by visual and proprioceptive sensations by prediction errors during the process of inference, as summarized in the previous figure. The resulting sequence of movements was configured to resemble handwriting and is shown as a function of location over time on the *lower right* (as *thick grey lines*). The *red dots* on these trajectories signify when a particular neuron or neuronal population encoding one of the hidden attractor states was active during *action* (*left panel*) and *observation* of the same action (*right panel*): More precisely, the dots indicate when responses exceeded half the maximum activity and are shown as a function of limb position. The *left panel* shows the responses during action and illustrates both a place-cell-like selectivity and directional selectivity for movement in an extrinsic frame of reference. The equivalent results on the right were obtained by presenting the same visual information to the agent but removing proprioceptive sensations. This can be considered as a simulation of action observation and mirror neuron-like activity

Action can also minimise surprise by minimising free energy or prediction errors. Neurobiologically, this is just saying that biological agents have reflexes—in the sense that they automatically minimise (proprioceptive) prediction errors. Formally, this corresponds to equipping a predictive coding scheme with classical reflex arcs—this is called as *active inference*. Put simply, agents will move in a way that they expect to move, so that top–down predictions become self-fulfilling prophecies and surprising exchanges with the world are avoided. These predictions can have a rich and dynamical structure. The example in Fig. 2 is based upon prior beliefs about visual and proprioceptive input that are realised by action to produce handwriting movements. These movements are driven by reflexes that fulfil predictions that the agent’s arm will be drawn to a succession of points that are prescribed by a high-level attractor or central pattern generator. Crucially, this sort of scheme lends itself not only to explaining itinerant motor behaviour—in terms of high-level attractors encoding prior beliefs—but also accommodates action observation of the sort associated with the mirror neuron system (Miall 2003; Rizzolatti and Craighero 2004).

## Sampling and agency

Hitherto, we have assumed that minimising sensory surprise or prediction errors is sufficient to minimise the entropy of the hidden states that cause sensations. As noted above, this rests on sampling sensory information that leaves little room for uncertainty about hidden states. However, we can relax this assumption if agents believe that they will sample sensations that minimise this uncertainty. In other words, one only has to believe that hidden states will disclose themselves efficiently and action will make these beliefs come true. This corresponds to sampling the world to maximise the posterior confidence in predictions. Crucially, placing prior beliefs about sampling in the perception–action cycle rests upon having a generative model that includes the effects of selective sampling. In other words, this sort of Bayes-optimal search calls on an internal model of how the environment is sampled. Implicit in a model of sampling is a representation or *sense of agency*, which extends active inference in an important way.

In summary, an imperative to maximise the posterior confidence about the causes of sensations emerges naturally from the basic premise that self-organising biological systems—like the brain—minimise the dispersion of their external states when immersed in an inconstant environment. This imperative—expressed in terms of prior beliefs how the world is sampled—is entirely consistent with the principle of maximum information transfer and formulations of salience in terms of Bayesian surprise (Barlow 1961; Bialek et al. 2001; Grossberg et al. 1997; Humphreys et al. 2009; Itti and Baldi 2009; Itti and Koch 2001; Olshausen and Field 1996; Optican and Richmond 1987). In what follows, we consider the neurobiological implementation of this prior belief, in the setting of visual search and *salience*: here, salience refers to the posterior confidence about the hidden causes of sensory input, as a function of where or how input is sampled.

## Modelling saccadic eye movements

To illustrate the sorts of behaviour that follow from the theoretical arguments above, we will look at visual searches and the control of saccadic eye movements. This treatment is based on four assumptions:

- The brain minimises the free energy of sensory inputs defined by a generative model.
- This model includes prior expectations that maximise salience.
- The generative model used by the brain is hierarchical, nonlinear and dynamic.
- Neuronal firing encodes posterior expectations about hidden states, under this model.

The first assumption is the free energy principle, which leads to active inference in the embodied context of action. The second assumption follows from need to minimise uncertainty about hidden states in the world. The third assumption is motivated easily by noting that the world is dynamic and nonlinear and that hierarchical causal structure emerges inevitably from a separation of temporal scales (Ginzburg and Landau 1950; Haken 1983). Finally, the fourth assumption is the Laplace assumption that—in terms of neural codes—leads to the *Laplace code* that is arguably the simplest and most flexible of all neural codes (Friston 2009).

Given these assumptions, one can simulate many neuronal processes by specifying a particular generative model. The resulting perception and action are specified completely by the above assumptions and can be implemented in a biologically plausible way; as described in many previous applications—see Table 1. In brief, the simulations in Table 1 use differential equations that minimise the free energy of sensory input using a generalised descent—see Fig. 1 and (Friston et al. 2010).

**Table 1:** Processes and paradigms that have been modelled using the active inference scheme in Eq. 1

Domain	Process or paradigm
Perception	Perceptual categorisation (bird songs) (Friston and Kiebel 2009a, b)
	Novelty and omission-related responses (Friston and Kiebel 2009a, b)
	Perceptual inference (speech) (Kiebel et al. 2009)
Sensory learning	Perceptual learning (mismatch negativity) (Friston and Kiebel 2009a, b)
Attention motor control	Attention and the Posner paradigm (Feldman and Friston 2010)
	Attention and biased competition (Feldman and Friston 2010)
	Retinal stabilization and oculomotor reflexes (Friston et al. 2010)
	Saccadic eye movements and cued reaching (Friston et al. 2010)
	Motor trajectories and place cells (Friston et al. 2011)
Sensorimotor integration	Bayes-optimal sensorimotor integration (Friston et al. 2010)
Behaviour	Heuristics and dynamical systems theory (Friston and Ao 2011)
	Goal-directed behaviour (Friston et al. 2009)
Action observation	Action observation and mirror neurons (Friston et al. 2011)

1 $$ \begin{aligned} \dot{\tilde{\mu }}(t) & = \mathcal{D}\tilde{\mu }(t) - \partial_{{\tilde{\mu }}} F(\tilde{s},\tilde{\mu }) \\ \dot{a}(t) & = - \partial_{a} F(\tilde{s},\tilde{\mu }) \\ \end{aligned} $$

**Fig. 3:**
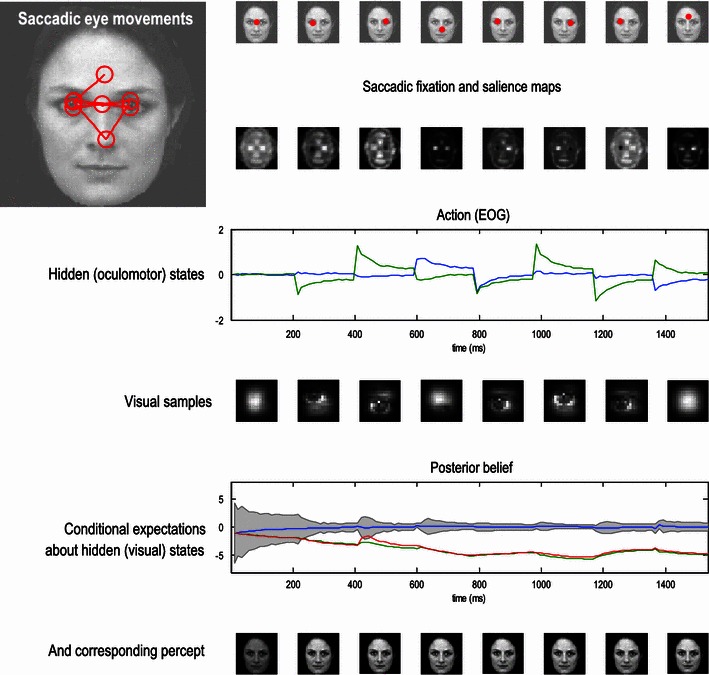
This figure shows the results of simulations in which a face was presented to an agent, whose responses were simulated using the active inference scheme described in the main text. In this simulation, the agent had three internal images or hypotheses about the stimuli it might sample (an upright face, an inverted face and a rotated face). The agent was presented with an upright face and its posterior expectations were evaluated over 16 (12 ms) time bins, until the next saccade was emitted. This was repeated for eight saccades. The ensuing eye movements are shown as *red dots* at the location (in extrinsic coordinates) at the end of each saccade in the upper row. The corresponding sequence of eye movements is shown in the insert on the *upper left*, where the *red circles* correspond roughly to the proportion of the image sampled. These saccades are driven by prior beliefs about the direction of gaze—based upon the saliency maps in the second row. Note that these maps change with successive saccades as posterior beliefs about the hidden states, including the stimulus, become progressively more confident. Note also that salience is depleted in locations that were foveated in the previous saccade. This reflects an inhibition of return that was built into the prior beliefs. The resulting posterior beliefs provide both visual and proprioceptive predictions that suppress visual prediction errors and drive eye movements, respectively. Oculomotor responses are shown in the third row in terms of the two hidden oculomotor states corresponding to vertical and horizontal displacements. The associated portions of the image sampled (at the end of each saccade) are shown in the fourth row. The final two rows show the posterior beliefs and inferred stimulus categories, respectively. The posterior beliefs are plotted in terms of posterior expectations and the 90 % confidence interval about the true stimulus. The key thing to note here is that the expectation about the true stimulus supervenes over its competing expectations and—as a result—posterior confidence about the stimulus category increases (the confidence intervals shrink to the expectation). This illustrates the nature of evidence accumulation when selecting a hypothesis or percept the best explains sensory data. Within-saccade accumulation is evident even during the initial fixation with further stepwise decreases in uncertainty as salient information is sampled by successive saccades

These coupled differential equations describe perception and action, respectively, and say that internal brain states—posterior expectations about hidden states—and action change in the direction that reduces free energy. The first is known as (generalised) predictive coding and has the same form as Bayesian (Kalman-Bucy) filters used in time series analysis; see also (Rao and Ballard 1999). The first term in Eq. (1) is a prediction based upon a time derivative operator. The second term—usually expressed as a mixture of prediction errors—ensures the changes in posterior expectations are Bayes-optimal predictions about hidden states of the world. The second differential equation says that action also minimises free energy—noting that free energy depends on action through sensory states. The differential equations in (1) are coupled because sensory input depends upon action, which depends upon perception through the posterior expectations. This circular dependency leads to a sampling of sensory input that is both predicted and predictable, thereby minimising free energy and surprise. To perform neuronal simulations it is only necessary to integrate or solve Eq. (1) to simulate the neuronal dynamics that encode posterior expectations and ensuing action. Figure 3 presents a simulation of saccadic eye movements, using prior expectations that lead to salient sampling. This is similar to the handwriting example in Fig. 2; however, eye movements are attracted not to points encoded by a central pattern generator but to locations that have the greatest salience. Here, salience is a function of location in visual space and reports the posterior confidence in current beliefs about the cause of sensory input that would be afforded by fictive sampling from that location.

The ensuing active inference can be regarded as a formal example of *active vision* (Wurtz et al. 2011)—sometimes described in enactivist terms as *visual palpation* (O’Regan and Noë 2001) and illustrates a number of key points. First, it discloses the nature of evidence accumulation in selecting a hypothesis or percept the best explains sensory data. Figure 3 shows that this proceeds over two timescales—within and between saccades. Within-saccade accumulation is evident even during the initial fixation, with further stepwise decreases in uncertainty as salient information is sampled. The within-saccade accumulation is formally related to evidence accumulation as described in models of perceptual discrimination (Gold and Shadlen 2003; Churchland et al. 2011). The transient changes in posterior expectations, shortly after each saccade, reflect the fact that new data are being generated as the eye sweeps towards its new target location. It is important to note that the agent is not just predicting visual input, but also how input changes with eye movements—this induces an increase in posterior uncertainty during the fast phase of the saccade. However, due to the veracity of the posterior beliefs, the posterior confidence shrinks again when the saccade reaches its target location. This shrinkage is usually to a smaller level than in the preceding saccade.

This illustrates the second key point, namely the circular causality that lies behind perception. Put simply, the only hypothesis that can endure over successive saccades is the one that correctly predicts the salient features that are sampled. This sampling depends upon action or an embodied inference that speaks directly to the notion of active vision and visual palpation (O’Regan and Noë 2001; Wurtz et al. 2011). This means that the hypothesis prescribes its own verification and can only survive if it is a correct representation of the world. If its salient features are not discovered, it will be discarded in favour of a better hypothesis. This provides a nice perspective on perception as hypothesis testing, where the emphasis is on the selective processes that underlie sequential testing. This is particularly pertinent when hypotheses can make predictions that are more extensive than the data that can be sampled at any one time.

## Conclusion

These simulations suggest that we can understand exploration of the sensorium in terms of optimality principles based on straightforward ergodic or allostatic principles. In other words, to maintain the constancy of our external milieu, it is sufficient to expose ourselves to predicted and predictable stimuli. Being able to predict what is currently seen also enables us to predict fictive sensations that we could experience from another viewpoint. Information theory suggests that the best viewpoint is the one that confirms our predictions with the greatest precision or certainty. In short, action fulfils our predictions, while we predict the consequences of our actions will maximise confidence in those predictions. This provides a principled way in which to explore and sample the world—for example, with visual searches using saccadic eye movements. These theoretical considerations are remarkably consistent with a number of compelling heuristics; most notably, the Infomax principle or the principle of minimum redundancy and recent formulations of salience in terms of Bayesian surprise.

In summary, we have tried to formalise the intuitive notion that our interactions with the world are akin to sensory experiments, by which we confirm our hypotheses about its causal structure in an optimal and efficient fashion. This mandates prior beliefs that the deployment of sensory epithelia and our physical relationship to the world will disclose its secrets—beliefs that are fulfilled by action. The resulting active or embodied inference means that not only can we regard perception as hypotheses, but we could regard action as performing experiments that confirm or disconfirm those hypotheses.
